# The inflammatory profile of cerebrospinal fluid, plasma, and saliva from patients with severe neuropathic pain and healthy controls-a pilot study

**DOI:** 10.1186/s12868-021-00608-5

**Published:** 2021-02-01

**Authors:** Mika Jönsson, Björn Gerdle, Bijar Ghafouri, Emmanuel Bäckryd

**Affiliations:** Pain and Rehabilitation Center, Department of Health, Medicine and Caring Sciences, Linköping University, Linköping, Sweden

**Keywords:** Biomarker, Cytokines, Inflammation, Neuroinflammation, Biofluids

## Abstract

**Background:**

Neuropathic pain (NeuP) is a complex, debilitating condition of the somatosensory system, where dysregulation between pro- and anti-inflammatory cytokines and chemokines are believed to play a pivotal role. As of date, there is no ubiquitously accepted diagnostic test for NeuP and current therapeutic interventions are lacking in efficacy. The aim of this study was to investigate the ability of three biofluids - saliva, plasma, and cerebrospinal fluid (CSF), to discriminate an inflammatory profile at a central, systemic, and peripheral level in NeuP patients compared to healthy controls.

**Methods:**

The concentrations of 71 cytokines, chemokines and growth factors in saliva, plasma, and CSF samples from 13 patients with peripheral NeuP and 13 healthy controls were analyzed using a multiplex-immunoassay based on an electrochemiluminescent detection method. The NeuP patients were recruited from a clinical trial of intrathecal bolus injection of ziconotide (ClinicalTrials.gov identifier NCT01373983). Multivariate data analysis (principal component analysis and orthogonal partial least square regression) was used to identify proteins significant for group discrimination and protein correlation to pain intensity. Proteins with variable influence of projection (VIP) value higher than 1 (combined with the jack-knifed confidence intervals in the coefficients plot not including zero) were considered significant.

**Results:**

We found 17 cytokines/chemokines that were significantly up- or down-regulated in NeuP patients compared to healthy controls. Of these 17 proteins, 8 were from saliva, 7 from plasma, and 2 from CSF samples. The correlation analysis showed that the most important proteins that correlated to pain intensity were found in plasma (VIP > 1).

**Conclusions:**

Investigation of the inflammatory profile of NeuP showed that most of the significant proteins for group separation were found in the less invasive biofluids of saliva and plasma. Within the NeuP patient group it was also seen that proteins in plasma had the highest correlation to pain intensity. These preliminary results indicate a potential for further biomarker research in the more easily accessible biofluids of saliva and plasma for chronic peripheral neuropathic pain where a combination of YKL-40 and MIP-1α in saliva might be of special interest for future studies that also include other non-neuropathic pain states.

## Introduction

Neuropathic pain (NeuP) is a complex chronic secondary pain condition characterized, according to ICD11, by spontaneous pain, allodynia, and hyperalgesia. It affects approximately 6.9–10% of the global population and represent a significant burden for patients and their families as well as society and healthcare systems [1–3].

The current definition of NeuP is pain caused by a lesion or disease of the somatosensory system, either centrally or peripherally [4]. Multiple etiological factors have been described in the development and maintenance of chronic NeuP including: neurodegenerative diseases, metabolic and autoimmune disorders, infections, neurotoxins, injuries, stroke, and cancer [5–7]. In addition to severe pain, many patients with NeuP also experience comorbidities such as anxiety, insomnia, depression etc. together with disability and reduced quality of life [4]. As such, NeuP represent a multidimensional entity with distinct subgroups of patients with particular sensory phenotypes and thus pathophysiological mechanisms [8].

Accordingly, the underlying molecular mechanisms of NeuP may vary between patients, which subsequently exemplifies the challenges associated with attaining accurate diagnosis and thus pharmacological interventions [1]. Consequently, research aiming to elucidate the molecular pathophysiological mechanisms associated with distinct somatosensory phenotypes of NeuP represent an important objective for current pain medicine. Several neural mechanisms have been implicated in NeuP, such as: ion channel alterations, pain message modulation and imbalances in excitatory and inhibitory somatosensory signaling. However, little is still known about the underlying pathophysiological mechanisms that initiates and maintains NeuP, and even less is known about the individual contribution of unique mechanisms for specific somatosensory phenotypes of NeuP [1, 9]. Indeed, the heterogeneity of neuropathic pain mechanisms, in combination with coexisting psychological aspects of chronic pain outlines the caveat of current pain medicine, which predominately targets the clinical symptoms rather than the causative factors [1, 5, 10].

Generally, chronic NeuP pain is mainly thought of as a disorder of the nervous system, including intricate mechanisms of neuronal excitability, ectopic discharge, central and peripheral sensitization. However, immune cells and their mediators have also been shown to act as key contributors to the instigation of various pain states; including NeuP [6, 11]. The extrinsic interactions between immune cells and neurons are both multidimensional and multifactorial, where reciprocal communication between neuronal and neuroinflammatory processes seems to play an essential role in the development and maintenance of chronic pain states [12, 13]. Particularly, 3 types of glial cells have been implicated in a complex temporal pattern of glial activation following peripheral nerve injury namely; astrocytes and microglia in the central nervous system (CNS) and satellite glial cells of the trigeminal ganglia and dorsal root ganglion in the peripheral nervous system (PNS) [14]. Notably, the peripheral immune response and central glial activation is linked through a complex plethora of neuronal and non-neuronal mechanisms, where cytokines and chemokines are thought to play a central modulatory role.

Cytokines belongs to a class of small molecules that are derived from immune cells and glial cells, which acts as pro- or anti-inflammatory mediators at picomolar and nanomolar concentrations to regulate synaptic activity as well as pain sensitivity [15, 16]. For instance, cytokines have been shown to modulate both excitatory and inhibitory synaptic transmissions at presynaptic-, postsynaptic and extrasynaptic sites [14]. As such, cytokines elicits a plethora of diverse mechanisms, extending from processes in the peripheral nervous system to the central nervous system as well as descending modulatory pathways [17]. It has been demonstrated in various animal models that cytokines can sensitize and directly activate nociceptors in the PNS thus giving rise to ectopic action potential discharges and thus contributing to peripheral sensitization [17]. Apart from giving rise to spontaneous action potential discharges, cytokines also modulate macrophages and the immune response and can de-sensitize the mu-opioid receptor [16]. Hence, cytokines are powerful glial modulators of synaptic transmissions that are potent at strikingly lower concentrations than regular neurotransmitters (GABA, glutamate, glycine etc.) that commonly function in micromolar concentrations [14]. As such, cytokines and chemokines are often viewed as the main link between the immune and nervous system, with mechanistically important functions in NeuP both within the CNS and PNS [18].

Hence, knowledge of the involvement of cytokines and chemokines in the neuro- immunological interaction in NeuP patients, occurring at different biofluid compartments, may aid in development of more effective pharmacological therapies. Accordingly, the aim of this study was to investigate the suitability of plasma, saliva, and CSF for studies of an inflammatory signature of cytokines and chemokines at a central, systemic, and peripheral level, collected concurrently from NeuP patients and healthy controls. To further explore the inflammatory profile of NeuP we also investigated if altered levels of inflammatory markers were correlated to pain intensity in neuropathic pain and if physiological quotients of cytokines in [saliva]/[plasma] and [plasma]/[CSF] differed between the groups.

## Materials and methods

### Patients

The cohort of neuropathic pain patients have been extensively described in a previous paper by Bäckryd et al. [19]. In short, patients were recruited from a clinical trial of intrathecal bolus injection of ziconotide, where CSF samples were collected before administration of the analgesic (ClinicalTrials.gov identifier NCT01373983). Inclusion criteria for participation were: (1) patient, ≥ 18 years of age, suffering from chronic (≥ 6 months) peripheral neuropathic pain resulting from trauma or surgery, with unsuccessful conventional pharmacological treatment; (2) average pain intensity last week according to a Visual Analogue Scale (VASPI) ≥ 40 mm [20]; (3) patient capable of judgment, i.e. able to understand information concerning the drug, the mode of administration, and evaluation of efficacy/side effects; [4] signed informed consent. Following informed consent, a medical examination was performed, and the following basic demographic data were registered; pain diagnosis; pain duration; present and past medical history; and concomitant medication. According to the criteria published by Treede et al., all patients had at least probable post-traumatic/post-surgical neuropathic pain [21]. Exclusion criteria and information about healthy controls have been published in detail elsewhere [19]. An overview of patients and healthy controls is presented in Table 1. Characteristics of the NeuP patients are listed in Table 2.

### Healthy controls

In brief, healthy controls were recruited by local advertisement at the Faculty of Medicine and Health Sciences, Linköping University Sweden, and by contacting healthy subjects from previous studies. Once, informed consent was obtained, absence of any significant medical condition was ensured by conducting a structured interview. Detailed information regarding healthy controls has been described previously and will not be discussed here [18].

### Ethics

The study was conducted in accordance with the Helsinki Declaration and Good Clinical Practice. The Ethical Review Board Regional Ethics Committee in Linköping approved the study (Dnr M136-06 and Dnr 2012/94 − 32). All participants received verbal and written information about the study, and after that, written informed consent was obtained from all the participants in this study.

**Table 1:** Overview of patients and healthy controls

Variables	Patients (n = 13)	Healthy controls (n = 13)	Statistics p-value
Age (years)	56 (39–65)	47 (22–57)	0.095
Sex (female/male)	6/7	8/5	0.440
Body mass index (kg/m^2^)	28.5 (20.2–32.4)	23.9 (19.5–28.4)	0.013

The data is shown as median (range) or percentage, with statistical comparisons between patients and healthy controls shown furthest to the right

**Table 2:** Neuropathic pain patient characteristics

Main cause of pain-ICD10	VASPI (0–100 mm)	Pain duration (months)	Comorbidities
S342 & G629	75	120	Alcohol dependency, tension headache, polyneuropathy, psoriasis
S549	64	300	None
S342	87	36	Hypertension, anemia, dyspepsia
S342	40	120	None
S342	78	79	None
S342	71	180	Diabetes, mild angina, autonomic neuropathy, panic anxiety
G629	68	78	None
S342	83	48	Localized bladder tumor
S142	58	18	None
S342	74	120	Depression
S142	84	18	Hypertension
S342	90	84	None
S841	94	52	None

S142—injury of nerve root of cervical spine

S342—injury of nerve root of lumbar and sacral spine

S549—injury o unspecified nerve at forearm level

G629—polyneuropathy, unspecified

VASPI—visual analogue scale for pain intensity last week

### Sample collection

#### Cerebrospinal fluid (CSF)

Intrathecal access was obtained by lumbar puncture with a 27 GA pencil-point Whitacre needle (BD, Franklin Lakes, NJ, USA) as described previously [22]. Briefly, a total sample of 10 mL CSF was draw from each subject by five syringes of 2 mL each.

#### Blood

Venous blood sample (10 ml) was collected in a EDTA tube.

#### Saliva

Whole saliva was collected using Salivette (Sarstedt). Subjects were not allowed to eat within 30 min of saliva collection. Subjects rinsed their mouth with water, and saliva sampling was collected after 10 min by putting a swab in the mouth for 3 min.

The samples were immediately cooled on ice and transported to Painomics® laboratory, Linköping University Hospital. Each sample was then centrifuged, divided into aliquots, and stored in − 70 °C awaiting analysis.

### Inflammatory profile analysis

The concentrations of 71 cytokines, chemokines and growth factors in saliva, plasma and CSF samples from patients and healthy controls was analyzed using a U-PLEX assay based on an electrochemiluminescent detection method (Meso Scale Diagnostics, Rockville, MD, USA) according to the manufacturer’s recommendations. Data were collected and analyzed using MESO QUICKPLEX SQ 120 instrument equipped with DISCOVERY WORKBENCH® data analysis software (Meso Scale Diagnostics, Rockville, MD, USA). The precision based on both intra and inter-assays variations were < 10 % within the detection limits provided by the manufacturer. Samples were thawed on the day of analysis, blinded to the clinical groupings and were randomly mixed. In short, 96-well plates were coated with linker-coupled capture antibodies (provided by the manufacturer) for one hour and then aspirated and washed with washing buffer (PBS/ 0.05 % Tween-20) 3 times. Standards and plasma samples (25 µl) were added to appropriate wells and incubated for one hour at room temperature with shaking. The fluid was then removed, and the wells were washed 3 times with washing buffer. Detection antibodies were added to each well, and incubated for 1 h at room temperature, followed by washing 3 times. After washing, 150 µl of reading buffer was added to each well. The plate was analyzed on the MSD instrument immediately. Standard curves were formed by fitting electrochemiluminescence signal from calibrators to a weighted 4-parameter logistic model. For the purposes of statistical analyses, any value that was below the lowest limit of detection (LLOD) for the assay was replaced with half of LLOD of the assay.

### Statistics

Statistical analyses were made using IBM SPSS (version 24.0; IBM Corporation, Route 100 Somers, New York, USA) and SIMCA-P+ (version 15.0; Sartorius Stedim Biotech, Umeå, Sweden) and *P* ≤ 0.05 was used as level of significance in all analyses. For descriptive statistics, all analyses were performed using IBM SPSS and the results were given as mean values. Mann-Whitney U test was used to compare groups. When calculating physiological quotients between saliva and plasma concentrations and plasma and CSF concentrations, all calculations were based on [saliva value]/[plasma value] and [plasma value]/[CSF value] respectively.

Traditional univariate statistical methods can quantify level changes of individual substances but disregard interrelationships and thereby ignore system-wide aspects. Moreover, traditional statistical methods (e.g., multiple, and logistic regression) are not designed to handle data sets with more variables than subjects (i.e., short, and broad data sets). Therefore, we used advanced multivariate data analysis by projection (MVDA) using SIMCA-P+. When applying MVDA, we followed the recommendations presented by Wheelock and Wheelock [23]. Variables were mean centred and scaled for unified variance (UV-scaling). The MVDA method of orthogonal partial least squares (OPLS) regression was used since the method can handle low subject-to-variable ratios of < 1, which is the common appearance of datasets in the omics field. Before OPLS analysis, the data was initially overviewed by unsupervised principal component analysis (PCA), which organizes and simplifies the data by separating relevant information from noise. Once PCA was conducted and potential multivariate outliers had been identified, OPLS discriminant analysis (OPLS-DA) was used to regress group discrimination, i.e. determining which cytokines/chemokines were important for class differences between patients and healthy controls. To measure the importance of each of the variables, the variable influence of projection (VIP) value was used, where VIP ≥ 1.0 (combined with the jack-knifed confidence intervals in the coefficients plot not including zero) were considered significant [19]. The OPLS-DA and OPLS were performed in two steps as described previously [24, 25]. The proteins with VIP ≥ 1 and absolute p(corr) > 0.5 from the first model were selected for a second new regression model and the new R^2^, Q^2^, and CV-ANOVA were presented in the results. The tables also present p(corr) for each significant molecule: the loading of each variable scaled as a correlation coefficient and thus standardizing the range from − 1 to + 1; p(corr) is stable during iterative variable selection and comparable between models. An absolute p(corr) > 0.4–0.5 is generally considered significant. R^2^ describes the goodness of fit – the fraction of sum of squares of all the variables explained by a principal component. Q^2^ describes the goodness of prediction – the fraction of the total variation of the variables that can be predicted by a principal component using cross validation methods. R^2^ should not be considerably greater than Q^2^; if R^2^ is substantially greater than Q^2^ (a difference > 0.3) [26] the robustness of the model is poor, implying overfitting [23]. Moreover, Analysis of Variance of Cross-Validated predictive residuals (CV-ANOVA), which is a SIMCA-P + diagnostic tool for assessing model reliability, was also computed. CV-ANOVA provides a familiar P-value metric for the model.

## Results

### Regression of class discriminating inflammatory substances

The inflammatory signature, at a central, systemic, and peripheral level, was investigated in 13 NeuP patients and 13 healthy controls. The data was initially controlled by conducting an unsupervised PCA to probe for potential multivariate outliers. No strong outliers were identified by Hotelling’s T2, and no serious moderate outliers by DModX (2 principal components, R^2^ = 0.25, Q^2^ = 0.02). Subsequently, an OPLS-DA regression model was computed to discriminate between patients and healthy controls (one latent variable (i.e., the predictive one), R^2^ = 0.503, Q^2^ = 0.413, P = 0.002 by CV-ANOVA).

**Fig. 1:**
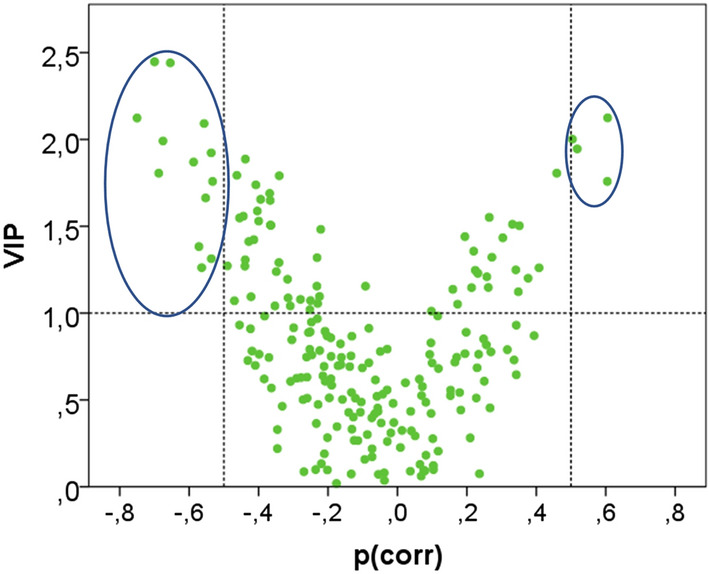
Volcano plot of cytokines/chemokines according to the OPLS-DA model. The x-axis shows p(corr) for each cytokine, where a negative p(corr) indicates higher levels in patients while a positive p(corr) indicates the opposite. The y-axis shows the variable importance of projection (VIP), which signifies the importance of each variable for the model. Cut-offs of p(corr) over 0.5 and under − 0.5, and VIP ≥ 1, were used and are illustrated by dotted lines. The 17 significant inflammatory proteins are highlighted within circles

Using a combination of absolute p(corr) > 0.5 and VIP > 1 as cut-offs, a total of 17 cytokines/chemokines were identified as significant for group separation (Fig. 1; Table 3). Thirteen of the 17 cytokines/chemokines were upregulated in NeuP patients, all of which came from plasma and saliva samples. The remaining 4 cytokines were downregulated in NeuP in the CSF and plasma samples. In total, 8 of the significant cytokines for group separation came from saliva, 7 from plasma and 2 from CSF samples. The glycoprotein YKL-40 was the only protein found to be significantly upregulated in two separate biofluids, plasma and saliva, in the NeuP cohort.

**Table 3:** Significant cytokines in respective biofluid identified by OPLS-DA

Cytokine/chemokine	Biofluid	Up or down regulated in patients	VIP	*p*(corr)
MIP-1α	Saliva	↑	2.52	− 0.75
IL-6	Plasma	↑	2.39	− 0.70
IL-1β	Saliva	↑	1.52	− 0.69
IL-1RA	Plasma	↑	2.31	− 0.68
TSLP	Saliva	↑	2.20	− 0.65
MIP-3β	Plasma	↑	2.00	− 0.59
MIP-1β	Saliva	↑	1.92	− 0.57
GRO-alpha	Saliva	↑	1.89	− 0.56
YKL-40	Plasma	↑	1.90	− 0.56
M-CSF	Saliva	↑	1.86	− 0.55
MCP-3	Plasma	↑	1.83	− 0.54
YKL-40	Saliva	↑	1.80	− 0.54
IL-18	Saliva	↑	1.79	− 0.53
CTACK	CSF	↓	1.72	0.50
IL-13	CSF	↓	1.77	0.52
ENA-78	Plasma	↓	2.07	0.60
GRO-alpha	Plasma	↓	2.07	0.61

Negative p(corr) indicates higher levels in patients while a positive p(corr) indicates the opposite. The cytokines are listed by descending order of importance in respect to absolute p(corr).

**Fig. 2:**
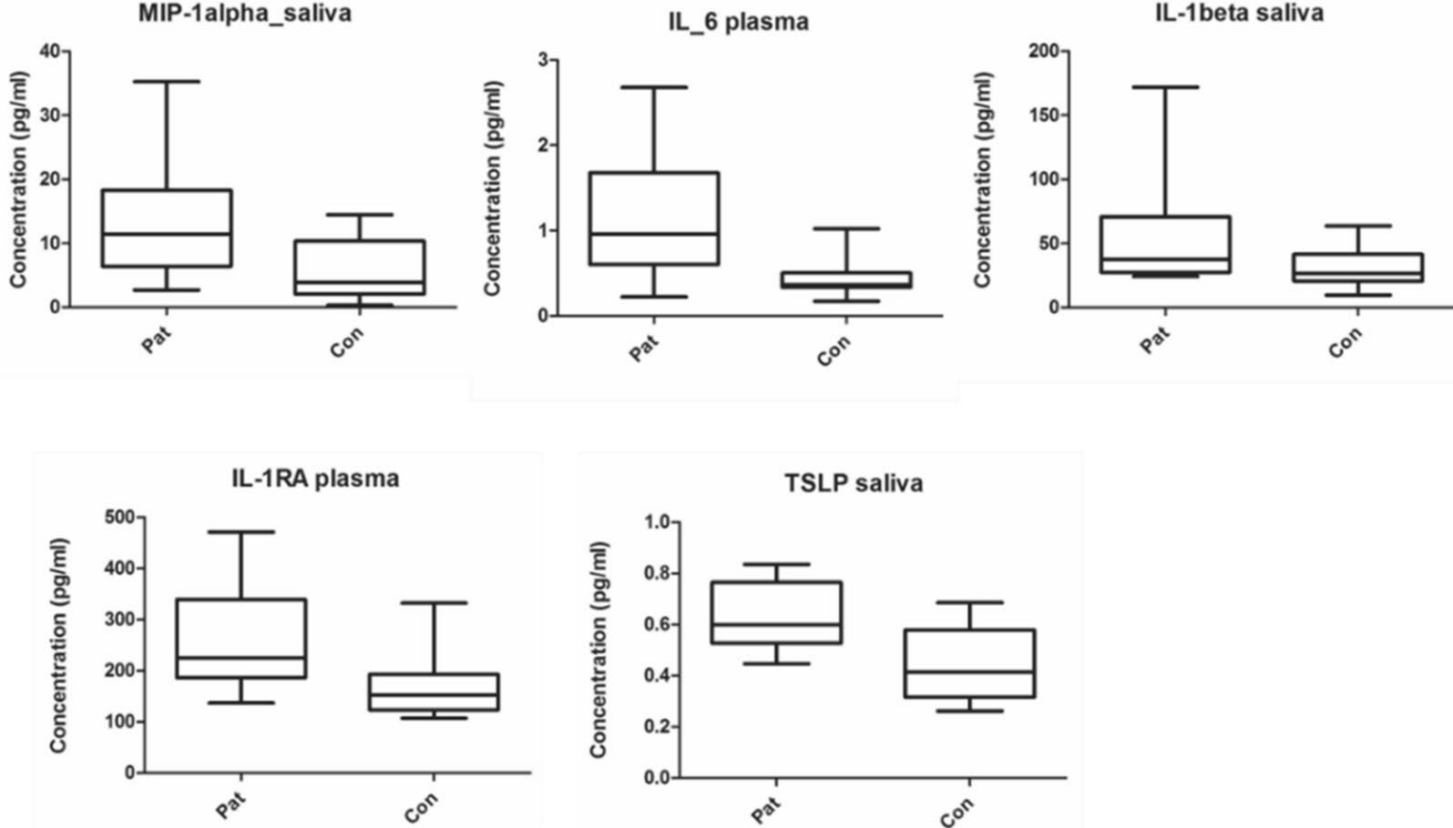
Box plots showing variations in cytokine concentration between patients and healthy controls for 5 inflammatory substances of significant value according to OPLS-DA. Median values are represented by horizontal lines, and the boxes represent the interquartile range. Minimum and maximum values are represented by the ends of the whiskers. MIP-1α (p = 0.017), IL-6 (p = 0.005), IL-1β (p = 0,043), IL-1RA (p = 0.008), and TSLP (p = 0.009)

From Table 3, five cytokines with the highest absolute p(corr) were selected (MIP-1α, IL-6, IL-1β, IL-1RA and TSLP). Three of the samples, MIP-1α, IL-β, and TSLP came from saliva samples whereas IL-6 and IL-1RA were taken from plasma samples. The concentration of each cytokine was compared between patients and healthy controls and differences were illustrated as box plots (Fig. 2).

### Regression of clinical pain parameters in respect to inflammatory signature

**Fig. 3:**
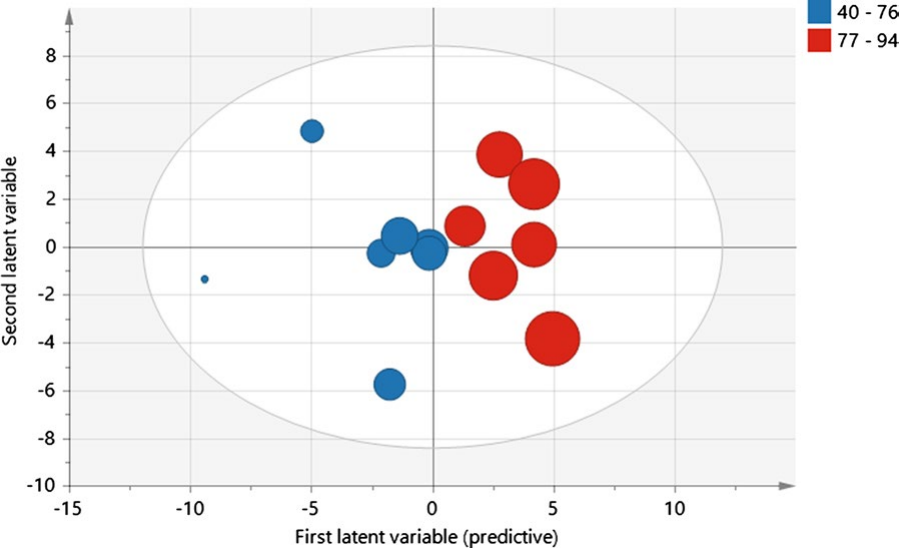
Score plot of OPLS regression model of pain intensity (VASPI). VASPI ranged from 40–94 and patients were dichotomized into moderate and high pain intensity (VASPI 40–76 respective 77–94). The model was significant according to CV-ANOVA (p = 0.041). Each dot represents a patient and the size of the dot indicates the importance for the model

To explore the inflammatory signature in respect to pain intensity in NeuP, an OPLS regression model was computed on the 13 NeuP patients to examine the association between VASPI (the Y-variable), and the 71 analyzed cytokines and chemokines in three biofluids (i.e., 213 X-variables, 2 latent variables, R^2^ = 0.961, Q^2^ = 0.675, and p = 0.041 by CV-ANOVA). VASPI ranged from 40 to 94 mm among the 13 NeuP patients, as shown in Table 2. The model is illustrated by a score plot (Fig. 3). A list of the contributing proteins important for VASPI is shown in Table 4. A total of 17 proteins had a VIP > 1, of which 15 were in plasma, 1 in CSF and 1 in saliva. Out of the 17 inflammatory substances important for VASPI, only 1 matched with the proteins presented in Table 3, namely ENA-78 (in plasma).

**Table 4:** Proteins important for OPLS regression of VASPI in patients

Cytokine/chemokine	Biofluid	VIP	p(corr)
IL-17D	Plasma	1.69	− 0.95
IL-17F	Plasma	1.61	− 0.92
IL-3	Plasma	1.55	− 0.87
IL-17E-IL-25	Plasma	1.5	− 0.85
IL-31	Plasma	1.51	− 0.85
IL-17B	Plasma	1.51	− 0.84
IL-23	Plasma	1.47	− 0.83
IL-33	Plasma	1.44	− 0.81
ENA-78	Plasma	1.34	− 0.72
VEGF-A	Plasma	1.26	− 0.70
IL-7	Plasma	1.28	− 0.68
GM-CSF	Plasma	1.19	− 0.67
MCP-2	Saliva	1.01	− 0.60
Fractalkine	Plasma	1.02	− 0.57
IL-6	CSF	1.01	0.56
MIP-5	Plasma	1.06	0.60
IL-17A-F	Plasma	1.08	0.62

Proteins are presented in descending order of importance with respect to absolute p(corr) of the first latent (predictive) variable. A positive p(corr) signifies a positive association with VASPI, and a negative p(corr) signifies the opposite

### Physiological quotient of cytokines/chemokines between biofluids

To further investigate the inflammatory profile of NeuP we calculated the physiological quotient of substances between [saliva]/[plasma] and [plasma]/[CSF] for patients and healthy controls. In Table 5, the physiological quotients between [saliva]/[plasma] and [plasma]/[CSF] of significant cytokines for group discrimination (Table 3) are presented. Similarly, in Table 6, the physiological quotients between [saliva]/[plasma] and [plasma]/[CSF] of inflammatory proteins important for VASPI (Table 4) are presented. A significant difference in [saliva]/[plasma] quotient between NeuP patients and healthy controls was seen for 2 proteins: MIP-1α (p = 0.039) and GRO-alpha (p = 0.024). For [plasma]/[CSF], a significant difference in quotients between groups was seen in 4 proteins; GRO-alpha (p = 0.015), IL- β (p = 0.029), IL-6 (p = 0.005), and YKL-40 (p = 0.022).

**Table 5:** Physiological quotients between [saliva]/[plasma] and [plasma]/[CSF] of significant cytokines for group discrimination

Cytokine/chemokine	[Saliva]/[plasma]			[Plasma]/[CSF]		
	Patient	Healthy control	Relative to controls	Patient	Healthy control	Relative to controls
MIP-1α	0.63 (0.15–1.38)	0.35 (0.02–0.98)	↑*	1.30 (0.78–1.86)	1.21 (0.79–1.89)	↑
IL-6	3.00 (0.15–21.23)	2.21 (0.11–10.40)	↑	1.47 (0.42–2.85)	0.74 (0.09–2.71)	↑*
IL-1β	502.12 (116.60-1764.62)	505.65 (53.26–1665.00)	↓	2.45 (0.48–5.50)	1.63 (0.24–10.82)	↑*
IL-1RA	0.38 (0.20–0.84)	0.40 (0.24–0.71)	↓	10.73 (6.03–24.88)	7.14 (4.20-12.43)	↑
TSLP	0.32 (0.18–0.47)	0.30 (0.12–0.61)	↑	1.55 (0.46–2.70)	1.78 (0.53–3.25)	↓
MIP-3β	0.01 (0.04–0.08)	0.06 (0.03–0.15)	↓	0.85 (0.17–1.90)	0.61 (0.23–1.02)	↑
MIP-1β	0.15 (0.03–0.38)	0.09 (0.02–0.22)	↑	3.70 (1.89–5.90)	3.90 (2.50–6.43)	↓
GRO-alpha	8.37 (1.45–44.80)	3.39 (0.18–22.15)	↑*	2.74 (0.98–8.96)	8.37 (0.79–28.08)	↓*
YKL-40	0.44 (0.11–0.70)	0.38 (0.04–0.74)	↑	0.85 (0.58–1.01)	0.74 (0.55–0.93)	↑*
M-CSF	3.88 (0.94–9.78)	2.56 (1.15–4.21)	↑	1.05 (0.80–1.38)	1.11 (0.62–1.75)	↓
MCP-3	1.22 (0.48–2.63)	1.97 (0.70–4.07)	↓	0.93 (0.59–1.23)	0.83 (0.68–1.07)	↑
IL-18	2.61 (0.20–8.46)	1.15 (0.21–3.09)	↑	122.99 (38.05-306.47)	104.26 (40.78-155.14)	↑
CTACK	0.00 (0.0008–0.005)	0.00 (0.00-0.01)	↑	122.22 (74.08-201.14)	106.56 (51.85–155.60)	↑
IL-13	0.46 (0.13–0.92)	0.59 (0.04–1.18)	↓	5.12 (1.34–11.21)	3.57 (1.25–13.99)	↑
ENA-78	0.06 (1.61–12.87)	3.01 (0.01–29.21)	↓	38.11 (2.20-141.85)	87.81 (2.98–327.60)	↓

Quotients for 15 out of 17 inflammatory substances presented in Table 3 are shown as 2 proteins, GRO-alpha and YKL-40, were presented twice in different biofluids. Data is presented as mean (range) where significant differences between groups (p < 0.05) are denoted with *, see "Regression of class discriminating inflammatory substances" section for exact figure

**Table 6:** Physiological quotients between [saliva]/[plasma] and [plasma]/[CSF] of inflammatory proteins important for VASPI

Cytokine/chemokine	[Saliva]/[plasma]			[Plasma]/[CSF]		
	Patients	Healthy controls	Relative to controls	Patients	Healthy controls	Relative to controls
IL-17D	4.57(0.95–8.71)	3.59 (0.92–6.48)	↑	0.07 (0.02–0.16)	0.10 (0.03–0.54)	↓
IL-17F	0.28 (0.02–0.76)	0.19 (0.04–0.77)	↑	14.72 (4.21–46.64)	21.24 (6.06–65.60)	↓
IL-3	3.74 (0.56-12-98)	3.06 (0.53-	↑	2.86 (0.39–19.03)	2.33 (0.36–8.99)	↑
IL-17E-IL-25	0.31 (0.09–0.59)	0.29 (0.01–1.53)	↑	25.74 (4.09-150.27)	40.80 (1.89-150.29)	↓
IL-31	0.24 (0.05–0.40)	0.25 (0.07–0.53)	↓	9.11 (2.86–21.59)	9.68 (1.86–17.31)	↓
IL-17B	7.27 (1.53–12.97)	6.84 (0.89–12.45)	↑	0.31 (0.11–1.68)	0.32 (0.06–0.98)	↓
IL-23	0.38 (0.02–2.73)	0.71 (0.04-7.00)	↓	92.23 (7.1-1027.13)	40.72 (3.43–110.70)	↑
IL-33	0.96 (0.34–2.30)	0.95 (0.20-0.3.06)	↑	2.57 (0.7-11.92)	2.59 (0.42–7.01)	↓
ENA-78	1.61 (0.06–12.87)	3.01 (0.01–29.21)	↓	38.11 (4.2-141.85)	87.81 (2.98–327.60)	↓
VEGF-A	72.43 (15.60-183.01)	40.97 (5.52–76.75)	↑	8.82 (3.95–21.58)	15.53 (4.59–77.95)	↓
IL-7	1.77 (0.59–5.08)	2.26 (0.38–6.11)	↓	4.58 (1.16–9.13)	6.21 (1.49–13.35)	↓
GM-CSF	25.00 (0.83-128.05)	15.31 (3.55–60.26)	↑	4.24 (0.42–23.21)	2.26 (0.64–5.28)	↑
I-309	0.90 (0.24–3.18)	2.19 (0.24–5.35)	↓	1.14 (0.81–1.92)	1.01 (0.69–1.25)	↑
GRO-alpha	8.37 (1.45–44.80)	3.39 (0.18–22.15)	↑*	2.74 (0.98–8.96)	8.37 (0.79–28.08)	↓*
IL-17A-F	0.48 (0.18–1.48)	4.17 (0.07–45.26)	↓	0.75 (0.47–1.39)	0.84 (0.32–1.35)	↓
MIP-5	0.01 (0.00-0.04)	0.01 (0.00-0.04)	↑	20.62 (13.38–37.51)	21.91 (12.96–34.69)	↓
MCP-2	0.16 (0.06–0.30)	0.19 (0.11–0.36)	↓	3.13 (1.58–6.55)	2.95 (2.14–3.95)	↑
Fractalkine	0.33 (0.07–0.97)	0.18 (0.04–0.38)	↑	3.90 (2.25–5.67)	3.91 (2.10–8.37)	↓
IL-6	3.00 (0.15–21.23)	2.21 (0.11–10.40)	↑	1.47 (0.42–2.85)	0.74 (0.09–2.71)	↑*

Data is presented as mean (range) where significant differences between groups (p < 0.05) are denoted with *, see "Regression of class discriminating inflammatory substances" for exact figure

## Discussion

Accumulating evidence indicates that the pathogenesis associated with neuropathic pain involves crosstalk between glial cells and neurons, where cytokine and chemokine networks play an important modulatory role [15]. In the present study, the inflammatory profile of patients with peripheral NeuP was investigated in three bio fluids; saliva, plasma, and CSF, using a U-PLEX assay based on an electro-chemiluminescent detection method. Out of a panel of 71 cytokines, chemokines, and growth factors, 17 proteins were found to be highly up- and down regulated in patients compared to healthy controls (Table 3). Comparably, when investigating the inflammatory signature of NeuP patients in respect to pain intensity, a total of 17 proteins were important for VASPI (VIP ≥ 1, p = 0.041 by CV-ANOVA) of which 15 were in plasma, 1 in CSF and 1 in saliva (Table 4).

The chemokine MIP-1α had the highest discriminatory power for group separation, as indicated by OPLS-DA, where the protein was found to be selectively up-regulated in saliva samples from patients suffering from peripheral NeuP. However, when investigating the clinical pain parameter VASPI, MIP-1α was not represented among the 17 proteins important for the OPLS regression of VASPI in patients. Moreover, when comparing the physiological quotient of MIP-1α between patients and healthy controls, there was a significant difference in the [saliva]/[plasma] quotient between groups (p = 0.039). MIP-1α in plasma did not differ between groups. This result support previous studies where low to moderate associations between cytokine concentrations in plasma and saliva has been demonstrated, which is believed to depend on the restrictive pathway of cytokines into the salivary glands [27, 28]. Consequently, the high concentration of MIP-1α in the saliva samples from patients does not appear to reflect systemic levels of the protein, but rather suggests the existence of increased local production. On the contrary, in another study where 48 cytokines, chemokines and growth factors were quantified in healthy human saliva, plasma and urine, MIP-1α was not detected in any of the samples [29]. This finding is contradictory to our data, where the chemokine was detected in all biofluids, both in patients and in healthy controls. The reasons behind the differences seen across studies could be due to differences in methods of analysis (where they used two multiplex bead-based kits) or perhaps because of the comparatively small sample size in each study.

Notably, whole saliva is not a single fluid, but rather a mixture of salivary gland secretions with different glands having varying contributions [30]. Salivary output and composition is highly intertwined with the autonomic nervous system (ANS), where the sympathetic system controls the serous aspect of the glands while the mucus part is under the influence of the sympathetic and parasympathetic system [31]. As such, salivation is a dynamic process which can be changed, both in composition, volume and flow, in response to several psychological states, such as stress or pain, both of which are present in NeuP [28]. However, it should also be noted that patients suffering from NeuP often use prescriptions where mouth dryness is a common side effect, thus presenting a confounding factor which could “concentrate” the saliva and hence cause biased cytokine/chemokine levels. It should be noted that the patients in this study were prescribed medicines as described elsewhere [19]. This is a major limitation concerning the internal validity of the saliva data in the present study.

Although there is limited literature on the involvement of MIP-1α in the pathogenesis of human neuropathic pain, there is evidence from animal models highlighting its putative implication. For instance, in the partial sciatic nerve ligation (PSL) model, long-lasting tactile allodynia and thermal hyperalgesia was associated with dramatic up-regulation of MIP-1α and its receptors (CCR1 and CCR5) on macrophages and Schwann cells of the injured sciatic nerve in mice [16]. Conversely, perineural injections of anti-MIP-1α prevented the induction of tactile allodynia and thermal hyperalgesia following PSL. In addition, the researchers also showed that recombinant MIP-1α could elicit both tactile allodynia and thermal hyperalgesia in the sham operated limb when it was injected perineurally and intraneurally [16]. Interestingly, IL-1β was also shown to be up-regulate in macrophages and Schwann cells of the injured sciatic nerve following PLS and that perineural injection of anti-IL-1β could prevent PLS-induced neuropathic pain. Moreover, this IL-1β up-regulation was inhibited by anti-MIP-1α, thus indicating a critical role of the MIP-1α in the pathogenesis of PLS-induced NeuP [16].

However, interpretations based on assumed analogies between animals and humans are fundamentally problematic and susceptible to incorrect assumptions when it comes to deciphering the complexity of human disease states [32]. For instance, murine MIP-1α is encoded by a single gene, whereas at least three genes (CCL3, CCL3-L1, and LD78γ) exist for human MIP-1α [33]. Of those, CCL3 and CCL3-L1 are transcribed and exist in variable copy numbers among individuals whereas LD78γ represents a pseudogene which is not encoded [33, 34]. Although, great sequence homology between CCL3 and CCL3-L1 (94 %), the isomeric proteins have biologically distinct functions [33, 35]. For instance CCL3-L1 is unlike CCL3 a CCR3 ligand, a more potent CCR5 agonist as well as a substrate for CD26 where it can be cleaved to have enhanced activity on both CCR1 and CCR5 [35]. Since, the activity of murine MIP-1α is more similar to human CCL3-L1 than CCL3, it has been suggested that CCL3-L1 and not CCL3 represents the true functional homologue of murine MIP-1α [35]. Yet, the CCL3 isoform has up till this point received far more attention in literature [34].

Interestingly, the extent of copy number variation of CCL3-L1 in a cohort of Caucasians have been described by Townson et al. where they also showed that LPS-stimulated monocyte-induced increase of CCL3-L1 copy number was related to an increased ratio of CCL3-L1-mRNA to CCL3-mRNA and hence functional protein [35]. Notably, 4 % of the examined individuals completely lacked any detectable CCL3-L1 and when this genotype was later investigated in a well-defined multiple sclerosis cohort, only 0.5 % of the individuals lacked CCL3-L1. As a result, the authors hypothesized that the genetic polymorphism seen in CCL3-L1 copy number could potentially have an impact on diseases where MIP-1α is known to be involved [35]. Thus, given that MIP-1α was significantly up-regulated in the saliva of patients with neuropathic pain, it would be interesting to extend the investigation to identify the relative isoform ratio of MIP-1α in NeuP conditions.

Following MIP-1α, the pro-inflammatory cytokine IL-6 showed the second highest discriminatory power between groups, where it was found to be selectively up-regulated in plasma samples from patients (Table 3). This supports previous findings done by our lab, where (using another method) plasma IL-6 levels showed a two-fold elevation in patients suffering from peripheral neuropathic pain, thus indicating systemic low-level inflammation among patients [36]. The up-regulation of IL-6 seen in plasma samples from patients was, however, not reflected in saliva or CSF samples (Table 3). Similarly, when looking at the physiological quotients there was a significant difference between patients and healthy controls for IL-6 [plasma]/[CSF], which was higher in patients (p = 0.005). This was however not seen in the [saliva]/[plasma] quotient (Table 5). The former is in line with previous research where salivary IL-6 and plasma levels of IL-6 have shown to be uncorrelated [37–40]. Thus our data supports previous suggestions that saliva does not provide an ideal measure of systemic IL-6 [37]. Notably, IL-6 has been shown to increase in both capillary and venous plasma in response to exercise, whereas regular exercise training leads to reduced circulating baseline levels of IL-6, both of which must be taken into consideration when analyzing the interleukin in plasma [37]. Given that chronic pain patients generally have low physical activity as a result from pain and/or comorbidities, it seems unlikely that the higher plasma IL-6 concentration depended on patients being more physically active than controls prior to having their blood drawn. On the contrary, it is possible that healthy controls had lower basal IL-6 levels if they exercised regularly; meaning that the difference in plasma IL-6 would rather reflect exercise habits than neuropathic pain.

Nonetheless, mounting evidence from animal studies indicate that pro-inflammatory cytokines, such as IL-6, are involved in both peripheral and central mechanisms of pain hypersensitivity. [41]. For instance, IL-6 has been shown influence nociceptor sensitization as well as enhancing translation in sensory neurons, thus affecting nociceptive plasticity [42–46]. IL-6 has also been implicated in mechanisms associated with central sensitization, where it has been shown to modulate inhibitory synaptic transmissions by reducing the frequency of spontaneous inhibitory postsynaptic currents in lamina II superficial dorsal horn neurons [41]. Interestingly, when investigating the clinical pain parameter VASPI, IL-6 in CSF was positively associated with VASPI, thus indicating that higher levels of IL-6 in CSF was related to higher pain intensity among the cohort of NeuP patients (Table 4).

IL-1β is another pro-inflammatory cytokine that has been extensively implicated in the pathogenesis of neuropathic pain [47]. For instance, the cytokine has been shown to modulate both excitatory and inhibitory synaptic transmissions in lamina dorsal horn neurons, thus suggesting a role in central sensitization [41]. Moreover, pharmacological studies on central administration of IL-1β has indicated that it can induce both analgesia and hyperalgesia depending on the brain region and on the dose injected [48]. In this study, IL-1β was significantly up-regulated in saliva taken from patients suffering from peripheral neuropathic pain compared to healthy controls. However, this up-regulation was not reflected in plasma or CSF samples of patients (Table 3). There was, however, a significant difference in the [plasma]/[CSF] quotient for IL-1β between patients and controls (p = 0.029), which was higher in patients. This was not seen in the [saliva]/[plasma] quotient between groups (Table 5). Noteworthy, saliva IL-1β levels were ~500x higher for both patients and controls compared to plasma levels (Table 5); hence, suggesting that IL-1β might be locally produced in the salivary glands.

Chitinase 3-like protein 1 (YKL-40) was the only protein, to be significantly up-regulated in two biofluids; its concentration was shown to be higher in both plasma samples and saliva samples taken from patients compared to healthy controls (Table 3). Unlike the 16 other cytokines, chemokines, and growth factors significant for group separation, YKL-40 was the only glycoprotein to be significant for this purpose. Likewise, there was a significant difference in [plasma]/[CSF] quotient between the groups, with higher levels of YKL-40 in patients (p = 0.022, Table 5).

YKL-40 is primarily secreted by chondrocytes, but it is also synthesized by macrophages, neutrophils, synoviocytes, cancer cells, vascular smooth muscle cells, and liver cells among others [49–51]. Not surprisingly, the glycoprotein possesses several biological functions. For instance, it has been suggested that YKL-40 may act as a matrix-degrading enzyme where it may modulate local inflammatory processes, cell proliferation, differentiation, stimulate angiogenesis, protect against apoptosis as well as playing a role in remodeling/degrading the extracellular matrix [52, 53]. Thus, the glycoprotein has been extensively investigated as a biomarker for several disease states such as cancer, type-2 diabetes, Alzheimer’s disease, cardiovascular diseases, arthritis, inflammatory bowel diseases etc. [51, 53–57]. To the best of our knowledge, this is the first time YKL-40 concentration has been shown to be elevated in two biofluids associated with peripheral neuropathic pain compared to healthy controls. However, YKL-40 levels in CSF was not shown to be significantly up-regulated in patients. It has previously been illustrated that YKL-40 levels in the cerebrospinal fluid, in the setting of CNS infection, appear to increase without a concomitant increase in serum levels, suggesting that YKL-40 produced in the brain does not influence the concentration seen in [plasma]/[serum] [51]. In accordance, our results address the reverse, suggesting that YKL-40 produced in the periphery does not appear to influence CSF levels, as this was not shown to be significantly up-regulated in patients in relation with plasma and saliva levels. Moreover, since the YKL-40 levels in saliva reflected systemic levels of the protein in plasma, we suggest that YKL-40 could potentially serve as a salivary biomarker for NeuP in combination with for example MIP-1α. However, whether up-regulated salivary YKL-40 and MIP-1α are specific for chronic neuropathic pain is yet to be determined and the level of each protein in saliva taken from patients with other non-neuropathic disease states needs to be investigated.

In contrast to YKL-40, the growth factor GRO-1α was the only protein to be significantly up- and downregulated in two separate biofluids. In saliva, GRO-1α was significantly upregulated whereas in plasma GRO-1α was significantly downregulated in patients compared to healthy controls (Table 3). Likewise, the physiological quotient [saliva]/[plasma] was significantly higher for patients compared to controls (p = 0.024). Conversely, for [plasma]/[CSF] the GRO-1α quotient was significantly lower for patients compared to healthy controls (p = 0.015, Table 5).

Unexpectedly, none of the 17 significant cytokines/chemokines detected for group separation was found to be significantly up-regulated in the cerebrospinal fluid among patients. However, 2 proteins, CTACK and IL-13, where elevated in CSF samples taken from healthy controls. Interestingly, peripheral IL-13 has been shown in animal models of neuropathic pain to reduce PLS-induced hyperalgesia by down-regulating IL-1β as well as reversing inflammatory macrophages and tactile allodynia [58, 59]. Thus, it is possible that IL-13 possess endogenous analgesic functions, which are down-regulated in conditions of chronic neuropathic pain.

### Limitations

There are several limitations in this study. One limitation was the relatively low number of included subjects (n = 26), which depended on the fact that this was an additional study conducted prior to a ziconotide trial and the number of patients were calculated with respect to outcomes in that trial. Another constraint concerned CSF sampling which is an invasive procedure that limited the inclusion of both gender and age matched healthy controls. Although the differences in age and gender were not statistically significant (p > 0.05) between the groups, it is worth mentioning that this might be due to the relatively low sample size. To be able to clarify if there are any association between the identified proteins and gender/age a larger cohort including only healthy controls without any diseases or NeuP are warranted. Furthermore, many of the significant cytokines in this study are known to be involved in a variety of different pathologies and the inflammatory profile may therefore not be specific for NeuP. It is possible that the cytokine profile presented indicates a state of chronic disease rather than a state of chronic neuropathic pain. Inclusion of patients with non-neuropathic chronic pain would have been an option to explore this specificity but then another potential confounding effect would have been introduced.

## Conclusions

There is much evidence from animal studies supporting a pivotal role of cytokines and chemokines in the crosstalk between neural and immunological systems associated with neuropathic pain. In this pilot study we investigated the inflammatory profile of patients with chronic peripheral NeuP in three separate biofluids, where 15 out of 17 proteins that were significant for group separation came from saliva and plasma samples. It was also shown that proteins in plasma samples had the highest correlation to pain intensity among the NeuP patients. These preliminary results indicate a potential for further biomarker research in the less invasive biofluids of saliva and plasma for chronic neuropathic pain, where a combination of YKL-40 and MIP-1α in saliva might be of special interest for future studies that also include other non-neuropathic pain states.

## Data Availability

The datasets generated and/or analyzed in this study are not publicly available as the Ethical Review Board has not approved the public availability of these data.
